# Supplementary material for: Partial Loss of FITM2 Function Causes Hereditary Spastic Paraplegia
Source: Hum Mutat. 2026 Oct 22;2026:5893503. doi: 10.1155/humu/5893503 (PMC13691184; doi:10.1155/humu/5893503)
Supplement: Supplementary file 1 [file HUMU-2026-5893503-s001.docx]

**Supplementary text 1**

**Molecular genetic analysis**

In both families, WES was performed on genomic DNA extracted from peripheral blood (QIAamp DNA Blood Kit) and captured with SureSelect V6+UTR-post technology (Agilent Technologies, Santa Clara, CA, USA). Sequencing was performed on an Illumina HiSeq 4000 sequencer (Illumina, San Diego, CA, USA) with 100 bp paired-end reads. Sequence reads were aligned to the GRCh37 (hg19) human genome reference. Read mapping, variant calling and variant annotation were performed using the software BWA-0.7.12, GATKv3.4.0 and SnpEff_v4.1g, respectively.

**Cryo-EM sample preparation and data acquisition**

The FIT2 protein was expressed in the human embryonic kidney (HEK)293S GnTI^-^ suspension cells (ATCC, RRID number: CVCL_A785), cultured in FreeStyle 293 Expression Medium (Thermo Fisher Scientific, 12338026) with 2% (v/v) FBS at 37°C under 8% CO_2_. When cell density reached ~2.5M mL^-1^, the pCMV14-FIT2-3xFLAG plasmids were transfected into the cells. For 1 liter of cell culture, 1 mg of plasmids were pre-mixed with 3 mg of linear polyethyleneimine (Polysciences, 23966) in 40 mL of Opti-MEM medium for 30 mins at room temperature before transfection. After 18 hours of shaking incubation at 37°C, 10 mM sodium butyrate was added, and the growth temperature was lowered to 30°C to boost protein expression. After 48 hours of treating sodium butyrate, the cells were harvested by centrifugation at 550 g and resuspended in lysis buffer (20 mM Tris, pH 8.0, 150 mM NaCl, 1 mM PMSF, 10% (v/v) glycerol) supplemented with cOmplete protease Inhibitor Cocktail tablet, EDTA-Free. Membranes were solubilized by adding 2% (w/v) GDN (Anatrace, GDN101) at 4°C by gentle agitation for 1 hour followed by centrifugation at 14,000 rpm for 1 hour to remove insoluble materials. The supernatant was subsequently incubated with anti-FLAG M2 resin (Sigma-Aldrich, A2220) for 1 hour. The resin was then packed onto a gravity-flow column (Bio-Rad, 7321010) and washed with 10 column volumes (CV) of buffer A (20 mM Tris pH 8, 150 mM NaCl, 0.02% (w/v) GDN). The FIT2 protein was eluted by 5CV of elution buffer (20 mM Tris, pH 8, 150 mM NaCl, 0.02% (w/v) GDN, 0.150 mg mL^-1^ 3xFLAG peptide). The eluted protein was concentrated and further purified by size-exclusion chromatography (SEC) on a Superdex 200 Increase column (Cytiva Life Science, 28-9909-44) equilibrated with buffer A. Peak fractions were pooled and concentrated to 8–10 mg mL^-1^ for cryo-EM analysis. For cryo-EM analysis, the concentrated protein was incubated with 3 mM Fos-Choline-8 (Anatrace, F300F). All grids were prepared with FEI Vitrobot Mark IV (FEI Thermo Fisher) at 4°C and 100% humidity. 3.5 µL of the sample was applied to fresh glow discharged Quantifoil R1.2/1.3, Cu 400 mesh (Electron Microscopy Sciences, Q4100CR1.3).

Cryo-EM data were collected with a Titan Krios microscope (FEI) operating at 300 keV equipped with a K3 summit direct electron detector (Gatan). Movie datasets were collected with SerialEM in super-resolution mode at 29,000x with a super-resolution pixel size of 0.413Å. Each movie contained 60 frames over 3 s exposure time, using a dose rate of ~15 e^-^Å^-2^s^-1^, resulting in a total dose of ~66 e^-^Å^-2^, and the defocus range was -0.6 to -1.7 μm.

A total of 4,058 and 4,545 super-resolution movies were collected. All two datasets were processed similarly. The movies were normalized and motion corrected using the patch-motion-correction algorithm by cryoSPARC (v4.4.0). Blob-based autopicking in cryoSPARC was implemented to select initial particles. After several rounds of 2D classification, selected particles were used for training in Topaz particle-picking pipeline. Particles picked by Topaz were subjected to several rounds of 2D classification for clean-up and selected final particles from each dataset (499,378 and 576,398 particles) were combined to perform the *ab initio* procedure in cryoSPARC. Three parallel *ab initio* reconstructions jobs were performed using identical settings, and particles from the class showing better features were selected from each job and combined for the next *ab initio* procedure. After several rounds of *ab initio* procedures, the final 95,285 particles were used for the acceptable FIT2 map with six transmembrane features.

**Plasmid construction**

pMB111-v1 and pMB111-v2 (expressing GFP-FIT2 H185-fs and GFP-FIT2 G100R, respectively) were derived from pMB111 (expressing GFP-FIT2 WT) via cloning of FIT2 mutant sequence using EcoRI-HF and XhoI sites. pMB65-v1 and pMB65-v2 (expressing GFP-FIT2 H185-fs and GFP-FIT2 G100R, respectively) were derived from mMB65 (expressing GFP-FIT2 WT) via cloning of FIT2 mutant sequence using AfeI and PmeI sites. pMB82-v1 and pMB-v2 (expressing FIT2 H185-fs-3xFLAG and FIT2 G100R-3xFLAG, respectively) were derived from mMB82 (expressing FIT2 WT-3xFLAG) via site-directed mutagenesis. For pMB82-v1, PCR primer pairs GACTTCCTCCCCAGAGCTGGAGAATCTGTACTTCCAG and CTGGAAGTACAGATTCTCCAGCTCTGGGGAGGAAGTC were used. For pMB82-v2, PCR primer pairs GCACCCTGCTTGTGCGCACGGCCATCTGGTAC and GTACCAGATGGCCGTGCGCACAAGCAGGGTGC were used. Mutagenized plasmids were checked by sequencing.

**Oleoyl-CoA flux measurement in microsomes**

Expi293F GnTI- suspension cells were transfected with plasmids pMB82 (FIT2 WT-3xFLAG), pMB82v1 (FIT2 H185-fs-3xFLAG) and, pMB82v2 (FIT2 G100R-3xFLAG) and, or pMB277 (FIT2 HHAA-3xFLAG) and 1 mg/ml polyethyleneimine (Polysciences, 23966). At 16 hours after transfection, 10 mM sodium butyrate (Sigma-Aldrich, 303410) was added to the cell culture, and at 48 hours after transfection, cells were harvested. The cell pellet was resuspended in lysis buffer (250 mM sucrose, 50 mM Tris-HCl, pH 7.4, 200 mM NaCl, 1 mM EDTA) supplemented with protease inhibitors (Roche). Cells were lysed using a Dounce homogenizer, and the cell lysates were centrifuged at 8,000 g for 10 min at 4°C to remove cell debris, followed by further centrifugation of the supernatant at 100,000 g for 60 min at 4°C to isolate microsomes. The pellet was resuspended in lysis buffer. For flux reactions, 100 µg of microsomes were mixed with reaction buffer (100 mM KAc, pH 7.0, 1 mM MgCl2, 20 mM NaCl, 0.625 mg/mL BSA) and adjusted to a final volume of 200 µL with lysis buffer. The assay was initiated through addition of 25 µM oleoyl-CoA containing [14C] oleoyl-CoA (American Radiolabeled Chemicals, 0527-50 µCi) as a tracer (0.1 µCi). Reactions were incubated at 37°C for 10 min and then stopped by addition of chloroform:methanol (2:1) and 2% phosphoric acid. The lower phase (containing lipid) was collected, dried under an air stream, resuspended in chloroform, and separated by TLC with a chloroform:methanol:water (65:25:4) solvent system to separate polar lipids. The TLC plates were exposed to a phosphor imager screen for 2 days and developed with the Typhoon FLA 7000 phosphor imager.

**Antibodies and immunoblotting**

Mammalian cell extracts were prepared for western blot analysis by lysing with lysis buffer (150 mM NaCl, 50 mM Tris-HCl, pH 7.4, 1% (v/v) Triton X-100 supplemented with a protease inhibitor cocktail), followed by centrifugation for 6 min at 16,000 g. The supernatant was denatured in Laemmli buffer at 37°C for 10 min. Protein extracts were separated on a 12% SDS-PAGE gel (Bio-Rad Laboratories) and transferred to a polyvinylidene difluoride membrane (Bio-Rad Laboratories). Yeast extracts were prepared for western blot analysis by lysing cells in 10% trichloroacetic acid, supplemented with protease inhibitor cocktail, bead vortexing at 4°C for 10 min, and centrifugation for 10 min at 16,000 g. The pellet containing precipitated protein was resuspended in Laemmli buffer containing β-mercaptoethanol and heated at 37°C for 10 min.

Cultured HDFs were lysed in RIPA buffer (Thermo Fisher Scientific, 89900), supplemented with EDTA-free protease inhibitor cocktail (Roche, 04693132001) and 1% (v/v) Triton X-100. After incubating for 1 hour, cells were spun for 30 min at 20,000 g. The supernatant was then denatured in Laemmli sample buffer (Bio-Rad, 1610747). Proteins were separated on a 4–15% SDS-PAGE gel (Bio-Rad, 4568084) and transferred to a nitrocellulose membrane (Thermo Fisher Scientific, IB23001).
