# Supplementary material for: Partial Loss of FITM2 Function Causes Hereditary Spastic Paraplegia
Source: Hum Mutat. 2026 Oct 22;2026:5893503. doi: 10.1155/humu/5893503 (PMC13691184; doi:10.1155/humu/5893503)
Supplement: Supplementary file 2 [file HUMU-2026-5893503-s002.docx]

**Supplementary text 2**

**Identification of FITM2 variants in a Greek ALS cohort**

**Patients and Methods**

The study cohort consisted of 353 Greek ALS cases from the biobank of the Neurogenetics Unit, 1^st^ Department of Neurology, National and Kapodistrian University of Athens, Eginition Hospital, Athens, Greece. Clinical diagnosis was based on established diagnostic criteria (Brooks et al. 2000). All cases gave written informed consent for the performance of molecular genetic testing, and the study was approved by the ethics committee of Eginition Hospital. Table 1 depicts clinical and demographic data of all 353 ALS patients screened for *FITM2* variants.

**Table 1**. Clinical and demographic characteristics of the Greek ALS cohort screened for *FITM2* variants

| **Variable** | **ALS cohort** |
| --- | --- |
| N | 353 |
| Gender, Male (%) | 224 (63.4) |
| Age at onset (years) | 58.8 ± 12.5 |
| Site of onset (%) |  |
| Bulbar | 88 (24.9) |
| Limb | 220 (62.3) |
| Bulbar and Limb | 14 (4.0) |
| Unknown | 31 (8.8) |
| FH, familial (%) | 24 (6.8) |
| ALS-FTD | 8 (2.3) |

Data are Mean ± SD; FH = family history; FTD = frontotemporal dementia; ALS = amyotrophic lateral sclerosis.

Whole genome sequencing (WGS) was performed in 353 Greek ALS patients at the New York Genome center (NYGC). WGS libraries were prepared using the Illumina TruSeq DNA PCR-free Library Preparation Kit in accordance with the manufacturer’s instructions. Briefly, 1ug of DNA was sheared using a Covaris LE220 sonicator. DNA fragments underwent end-repair, bead-based size selection, adenylation, and Illumina sequencing adapter ligation. Paired-end 150bp reads were aligned to the GRCh38 human reference using the Burrows-Wheeler Aligner (BWA-MEM v0.7.15) and processed using the GATK best-practices workflow that includes marking of duplicate reads by the use of Picard tools (v2.4.1, http://picard.sourceforge.net), and base quality score recalibration (BQSR) via Genome Analysis Toolkit (GATK v3.5) (PMID: 20644199, 21478889). HaplotypeCaller was run on each sample separately in GVCF mode (GATK v3.5). This produced a file format called gVCF (for genomic VCF). GVCFs were then run through GATK v3.5 to produce separate VCF files for all samples.

The VCF files were downstream processed at the Greek Genome Centre of the Biomedical Research Foundation of the Academy of Athens (BRFAA). WGS data from all the samples were screened for variants in the *FITM2* gene (OMIM®: 612029). Firstly, the whole gene locus was captured and then variants were selected from all 353 VCF files. Annotation was performed using Annovar build hg38 (refGene, gnomad211_exome, clinvar_latest, oncokb_43, avsnp150, cosmic_coding, intervar_latest, dbnsfp42c, dbscsnv11).

Variant filtering and prediction were performed at Neurogenetics Unit, 1^st^ Department of Neurology, National and Kapodistrian University of Athens using VarAFT (Version 2.17-1) and Annovar. All exonic variants were cross-checked per clinical significance and pathogenicity scores using ClinVar and Varsome, respectively. Finally, they were classified using the ACMG-AMP criteria (Richards et al. 2015).

**Results**

Forty-one *FITM2* exonic variants were identified in total in our ALS cohort (Table 2). All likely pathogenic and VUS variants were detected in exon 2. One likely pathogenic variant (c.538C>T) was detected in a 63-year-old woman with lower limb-onset ALS at 61 years, who died at the age of 66 as a result of respiratory failure. She was the mother of the two affected siblings of the second Greek family and has been described in more detail elsewhere in the manuscript. Interestingly, the VUS variant identified *in-trans* with causative variants in both Greek families (c.298G>C) was detected in a 73-year-old man with sporadic limb-onset ALS at the age of 72. A further VUS variant (c.598T>A) was detected in two patients from our cohort. The first one was a 75-year-old man with bulbar-onset sporadic ALS at the age of 71. The second one was a 72 year-old-man with bulbar-onset at the age of 70. Finally, a VUS variant (c.353C>T) was detected in a 65-year-old woman with bulbar-onset sporadic ALS at the age of 64.

**Table 2.** *FITM2* variants detected in Greek ALS cohort.

| **Variant ID (rs)** | **Nucleotide change** | **AA change** | **Exon** | **Prediction** | **No of cases** |
| --- | --- | --- | --- | --- | --- |
| NA | c.36G>A | p.R12R | Exon 1 | Benign-likely benign | 1 |
| rs148377517 | c.96G>A | p.M32I | Exon 1 | Benign-likely benign | 31 |
| rs147548991 | c.252C>G | p.T84T | Exon 2 | Benign-likely benign | 1 |
| rs754943445 | c.340A>G | p.I114V | Exon 2 | Benign-likely benign | 2 |
| rs990943873 | c.354G>A | p.T118T | Exon 2 | Benign-likely benign | 1 |
| rs750030975 | c.298G>C | p.G100R | Exon 2 | VUS | 1 |
| rs147373369 | c.353C>T | p.T118M | Exon 2 | VUS | 1 |
| rs1038354869 | c.598T>A | p.F200I | Exon 2 | VUS | 2 |
| NA | c.538C>T | p.R180X | Exon 2 | Likely pathogenic | 1 |
| Total variants identified in exonic regions of *FITM2* gene | | | | | 41 |

NA = not available; VUS = variant of uncertain significance.
