# Supplementary material for: Partial Loss of FITM2 Function Causes Hereditary Spastic Paraplegia
Source: Hum Mutat. 2026 Oct 22;2026:5893503. doi: 10.1155/humu/5893503 (PMC13691184; doi:10.1155/humu/5893503)
Supplement: Supplementary file 3 [file HUMU-2026-5893503-s007.zip › Figure S7.pdf]

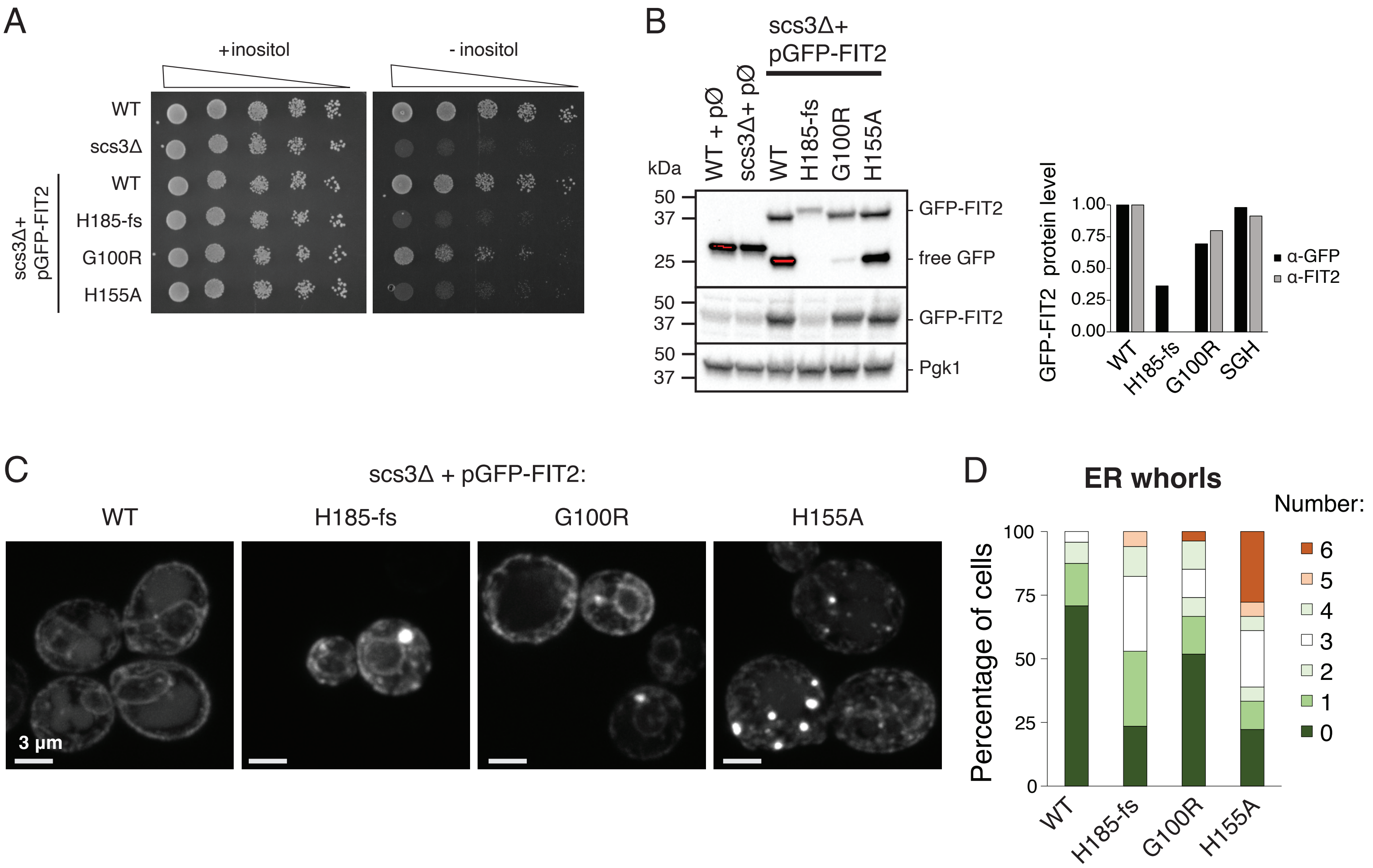
A
+inositol
- inositol
WT
scs3Δ
scs3Δ+ pGFP-FIT2
WT
H185-fs
G100R
H155A
B
scs3Δ+ pGFP-FIT2
WT + pØ
scs3Δ+ pØ
WT
H185-fs
G100R
H155A
kDa
50
37
25
GFP-FIT2
free GFP
GFP-FIT2
Pgk1
GFP-FIT2 protein level
1.00
0.75
0.50
0.25
0.00
α-GFP
α-FIT2
WT
H185-fs
G100R
SGH
C
scs3Δ + pGFP-FIT2:
WT
H185-fs
G100R
H155A
3 µm
D
ER whorls
Number:
6
5
4
3
2
1
0
Percentage of cells
100
75
50
25
0
WT
H185-fs
G100R
H155A
