# Supplementary material for: Partial Loss of FITM2 Function Causes Hereditary Spastic Paraplegia
Source: Hum Mutat. 2026 Oct 22;2026:5893503. doi: 10.1155/humu/5893503 (PMC13691184; doi:10.1155/humu/5893503)
Supplement: Supplementary file 3 [file HUMU-2026-5893503-s007.zip › Figure S6.pdf]

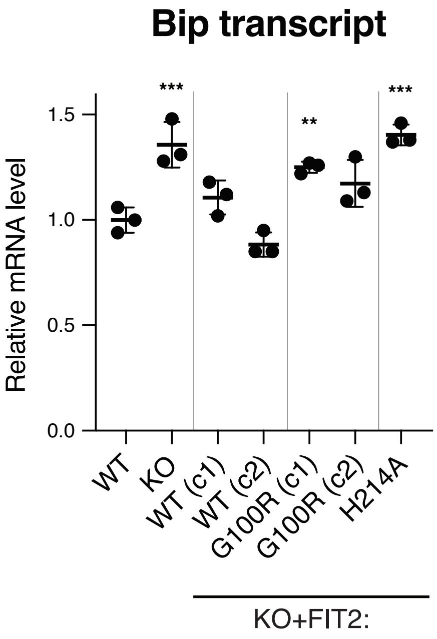

Bip transcript
Relative mRNA level
0.0
0.5
1.0
1.5
***
**
***
WT
KO
WT (c1)
WT (c2)
G100R (c1)
G100R (c2)
H214A
KO+FIT2:

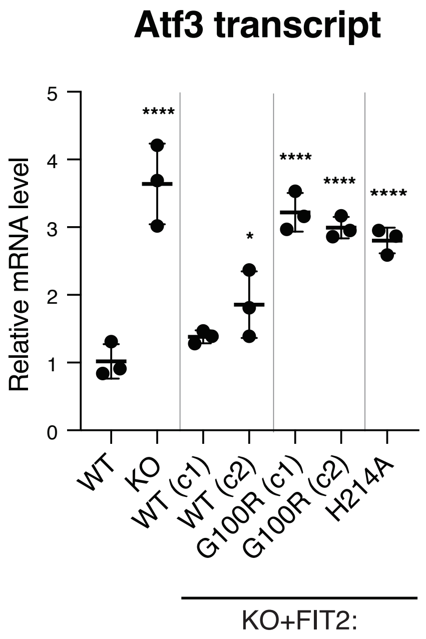

Atf3 transcript
Relative mRNA level
0
1
2
3
4
5
****
*
****
****
****
WT
KO
WT (c1)
WT (c2)
G100R (c1)
G100R (c2)
H214A
KO+FIT2:
