# Supplementary material for: Partial Loss of FITM2 Function Causes Hereditary Spastic Paraplegia
Source: Hum Mutat. 2026 Oct 22;2026:5893503. doi: 10.1155/humu/5893503 (PMC13691184; doi:10.1155/humu/5893503)
Supplement: Supplementary file 3 [file HUMU-2026-5893503-s007.zip › Figure S5.pdf]

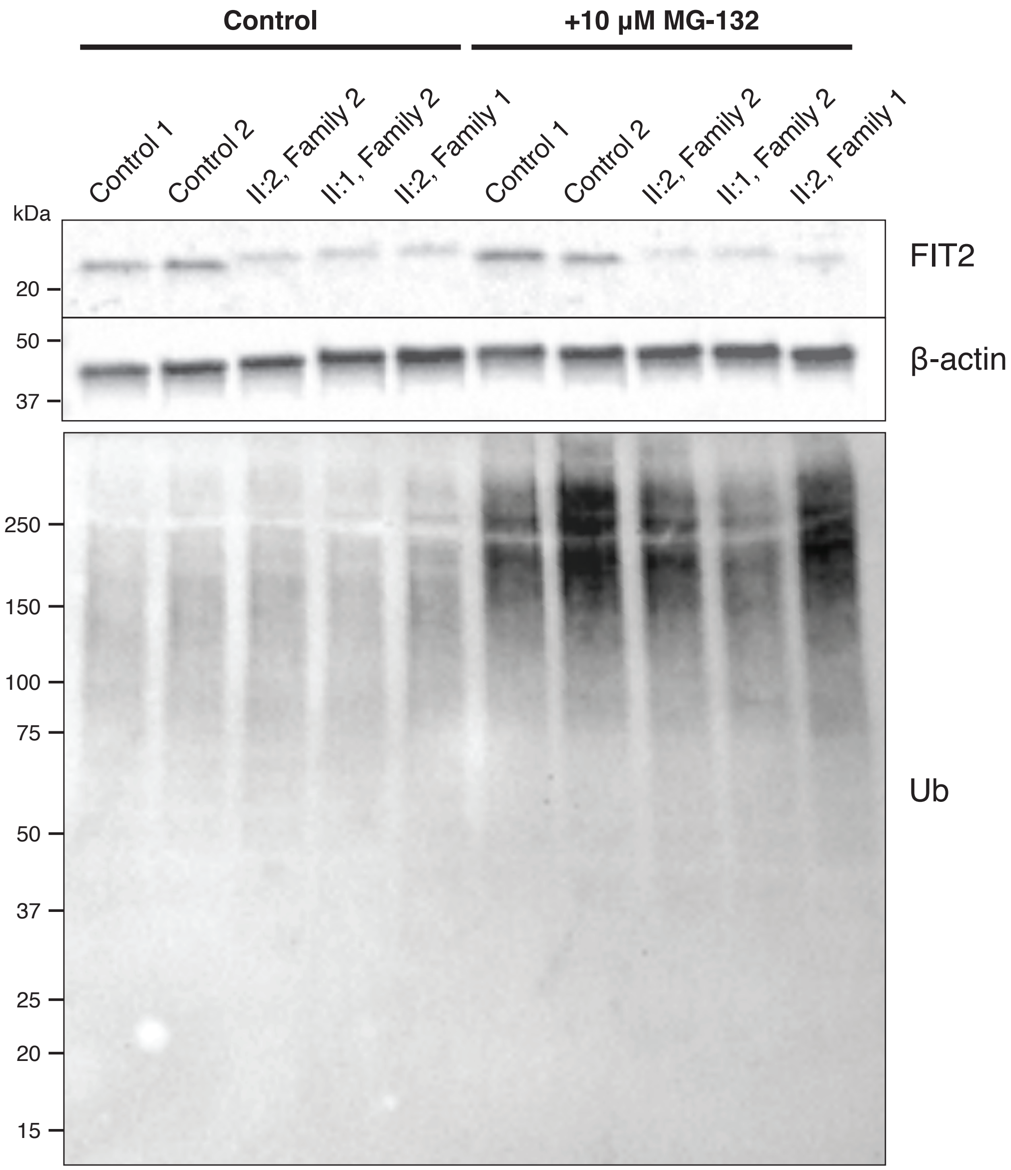
Control
+10 μM MG-132
Control 1
Control 2
II:2, Family 2
II:1, Family 2
II:2, Family 1
Control 1
Control 2
II:2, Family 2
II:1, Family 2
II:2, Family 1
kDa
20
50
37
250
150
100
75
50
37
25
20
15
FIT2
β-actin
Ub
