# Supplementary material for: Partial Loss of FITM2 Function Causes Hereditary Spastic Paraplegia
Source: Hum Mutat. 2026 Oct 22;2026:5893503. doi: 10.1155/humu/5893503 (PMC13691184; doi:10.1155/humu/5893503)
Supplement: Supplementary file 3 [file HUMU-2026-5893503-s007.zip › Figure S4.pdf]

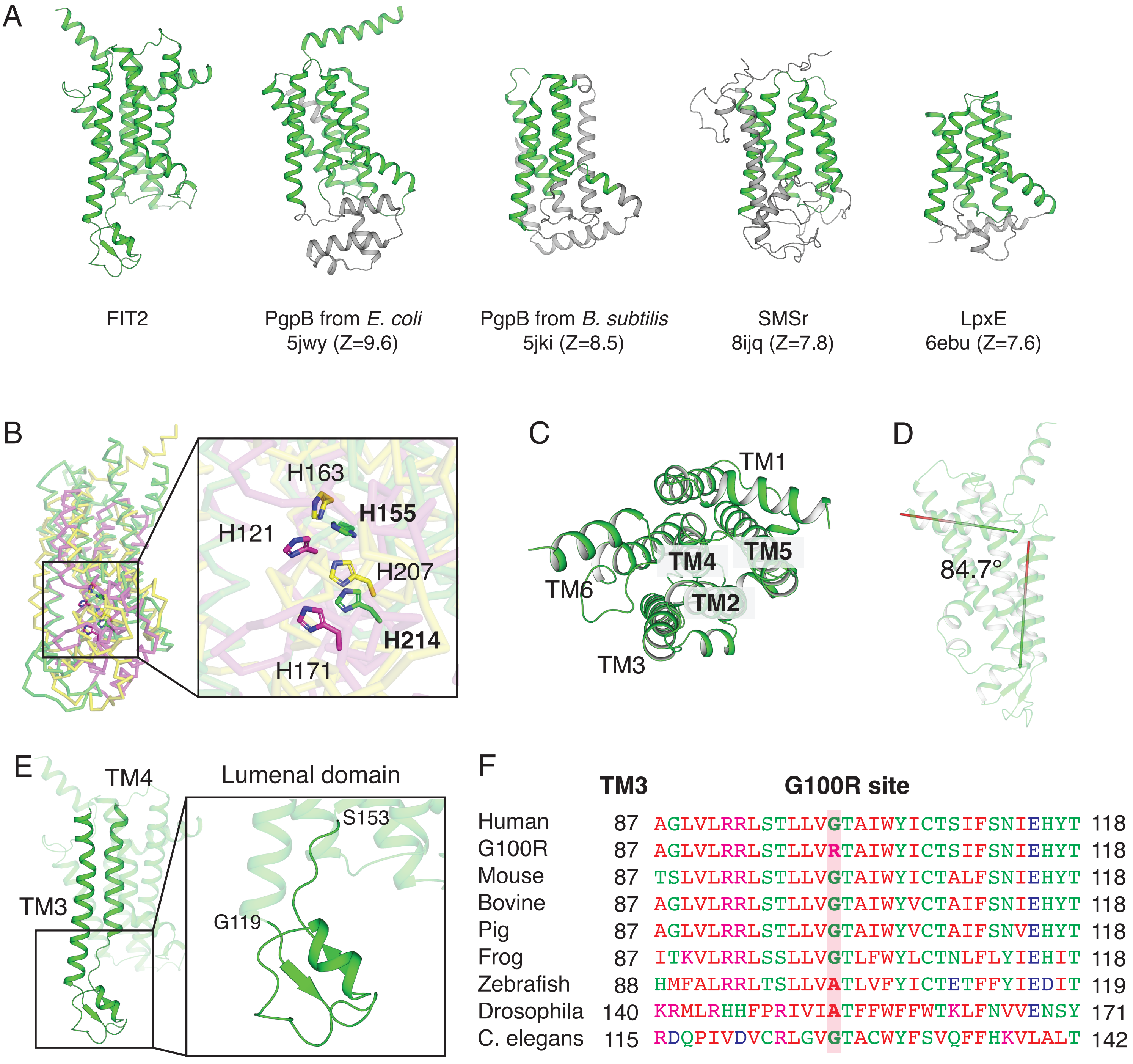
A
FIT2
PgpB from E. coli
5jwy (Z=9.6)
PgpB from B. subtilis
5jki (Z=8.5)
SMSr
8ijq (Z=7.8)
LpxE
6ebu (Z=7.6)
B
H163
H155
H121
H207
H214
H171
C
TM1
TM4
TM5
TM6
TM2
TM3
D
84.7°
E
TM4
TM3
Lumenal domain
S153
G119
F
TM3
G100R site
Human 87 AGLVLRRLSTLLVGTAIWYICTSIFSNIEHYT 118
G100R 87 AGLVLRRLSTLLVRTAIWYICTSIFSNIEHYT 118
Mouse 87 TSLVLRRLSTLLVGTAIWYICTALFSNIEHYT 118
Bovine 87 AGLVLRRLSTLLVGTAIWYVCTAIFSNIEHYT 118
Pig 87 AGLVLRRLSTLLVGTAIWYVCTAIFSNVEHYT 118
Frog 87 ITKVLRRLSSLLVGTLFWYLCTNLFLYIEHIT 118
Zebrafish 88 HMFALRRLTSLLVATLVFYICTETFFYIEDIT 119
Drosophila 140 KRMLRHHFPRIVIATFFWFFWTKLFNVVENSY 171
C. elegans 115 RDQPIVDVCRLGVGTACWYFSVQFFHKVLALT 142
