# Supplementary material for: Partial Loss of FITM2 Function Causes Hereditary Spastic Paraplegia
Source: Hum Mutat. 2026 Oct 22;2026:5893503. doi: 10.1155/humu/5893503 (PMC13691184; doi:10.1155/humu/5893503)
Supplement: Supplementary file 3 [file HUMU-2026-5893503-s007.zip › Figure S3.pdf]

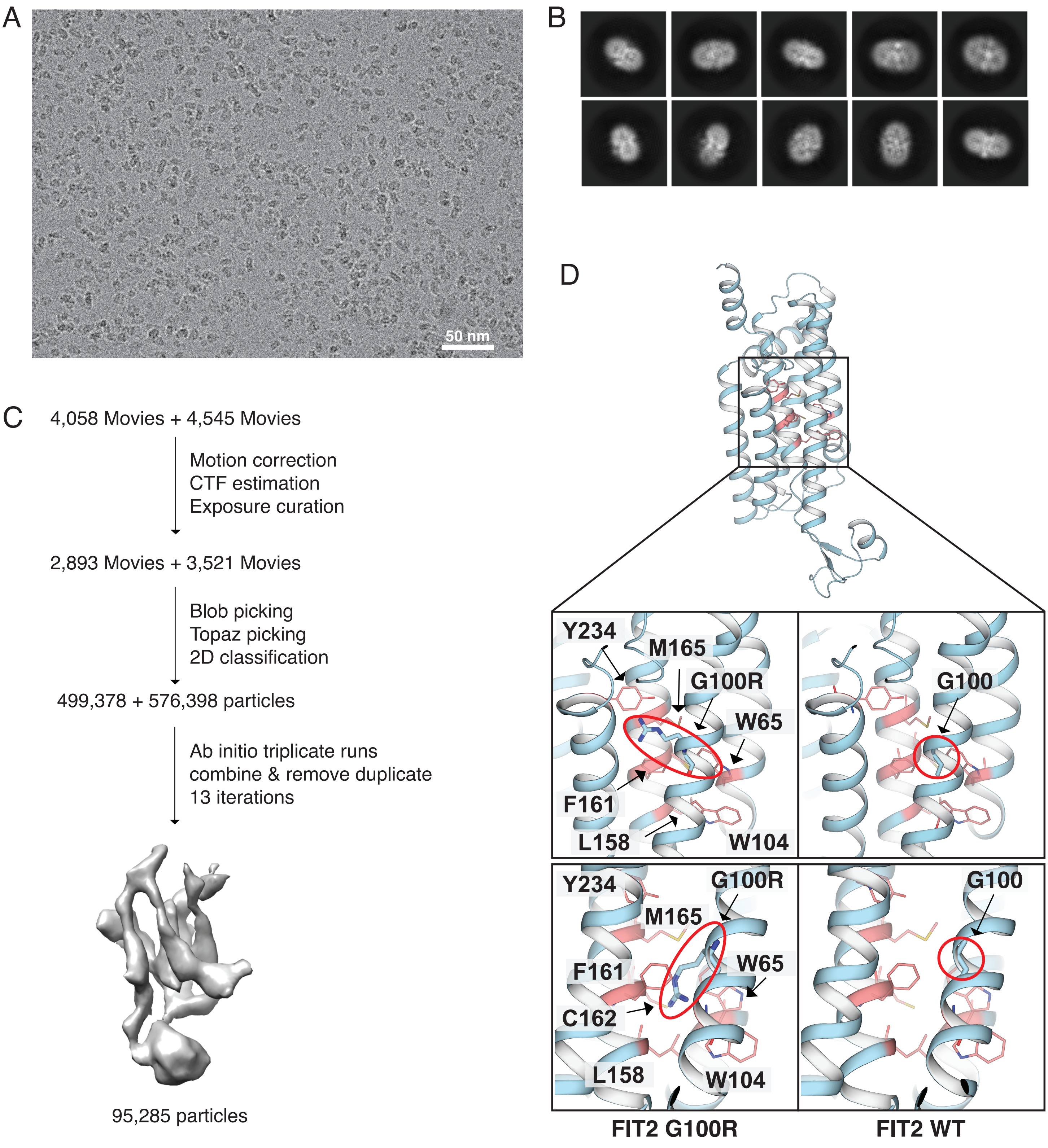
A
50 nm
B
C
4,058 Movies + 4,545 Movies
Motion correction
CTF estimation
Exposure curation
2,893 Movies + 3,521 Movies
Blob picking
Topaz picking
2D classification
499,378 + 576,398 particles
Ab initio triplicate runs
combine & remove duplicate
13 iterations
95,285 particles
D
Y234
M165
G100R
W65
F161
L158
W104
G100
Y234
G100R
M165
F161
W65
C162
L158
W104
G100
FIT2 G100R
FIT2 WT
