# Supplementary material for: Partial Loss of FITM2 Function Causes Hereditary Spastic Paraplegia
Source: Hum Mutat. 2026 Oct 22;2026:5893503. doi: 10.1155/humu/5893503 (PMC13691184; doi:10.1155/humu/5893503)
Supplement: Supplementary file 3 [file HUMU-2026-5893503-s007.zip › Figure S2.pdf]

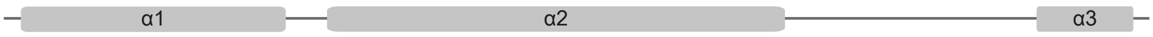


```
WT        MEHLERCEWLLRGTLVRAAVRRYLPWALVASMLAGSLLKELSPLPESYLSNKRNVLNVYF  60
H185-fs   MEHLERCEWLLRGTLVRAAVRRYLPWALVASMLAGSLLKELSPLPESYLSNKRNVLNVYF  60
R180-Ter  MEHLERCEWLLRGTLVRAAVRRYLPWALVASMLAGSLLKELSPLPESYLSNKRNVLNVYF  60
G100R     MEHLERCEWLLRGTLVRAAVRRYLPWALVASMLAGSLLKELSPLPESYLSNKRNVLNVYF  60
          ************************************************************
```

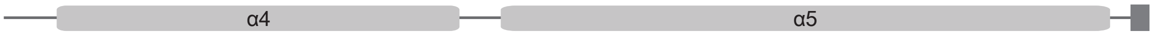


```
WT        VKVAWAWTFCLLLPFIALTNYHLTGKAGLVLRRLSTLLVGTAIWYICTSIFSNIEHYTGS  120
H185-fs   VKVAWAWTFCLLLPFIALTNYHLTGKAGLVLRRLSTLLVGTAIWYICTSIFSNIEHYTGS  120
R180-Ter  VKVAWAWTFCLLLPFIALTNYHLTGKAGLVLRRLSTLLVGTAIWYICTSIFSNIEHYTGS  120
G100R     VKVAWAWTFCLLLPFIALTNYHLTGKAGLVLRRLSTLLVRTAIWYICTSIFSNIEHYTGS  120
          *************************************** ********************
```

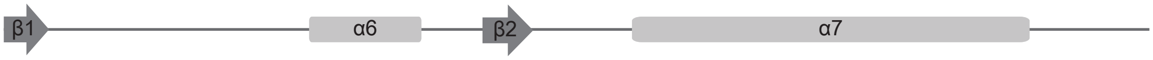


```
WT        CYQSPALEGVRKEHQSKQQCHQEGGFWHGFDISGHSFLLTFCALMIVEEMSVLHEVKTDR  180
H185-fs   CYQSPALEGVRKEHQSKQQCHQEGGFWHGFDISGHSFLLTFCALMIVEEMSVLHEVKTDR  180
R180-Ter  CYQSPALEGVRKEHQSKQQCHQEGGFWHGFDISGHSFLLTFCALMIVEEMSVLHEVKTD-  179
G100R     CYQSPALEGVRKEHQSKQQCHQEGGFWHGFDISGHSFLLTFCALMIVEEMSVLHEVKTDR  180
          ***********************************************************
```

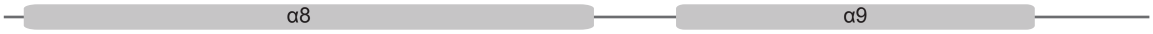


```
WT        SHCLHTAITTLVVALGILTFIWVLMFLCTAVYFHNLSQKVFGTLFGLLSWYGTYGFWYPK  240
H185-fs   SHCLLPPHRHHHPGCGPGHSDFHLGVDVSVHSCLFPQLVPEGVWHLVWFAELVRDIRVLV  240
R180-Ter  ------------------------------------------------------------
G100R     SHCLHTAITTLVVALGILTFIWVLMFLCTAVYFHNLSQKVFGTLFGLLSWYGTYGFWYPK  240
```

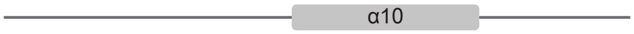


```
WT        GFWYPKAFSPGLPPQSCSLNLKQDSYKK-----  262
H185-fs   SESLFPRTSSPEL--------------------  253
R180-Ter  ---------------------------------
G100R     GFWYPKAFSPGLPPQSCSLNLKQDSYKK-----  262
```
